# Remineralization effect of Er;Cr:YSGG laser irradiation with or without acidulated phosphate fluoride application on deciduous teeth enamel surface with induced white spot lesion. An *in vitro* study

**DOI:** 10.4317/jced.61561

**Published:** 2024-06-01

**Authors:** Saba-Amer-Abed Mahdi, Basima-Mohammed-Ali Hussein

**Affiliations:** 1 University of Baghdad, Institute of Laser for Postgraduate Studies, Baghdad, Iraq; 2 Assist. Prof. Dr. University of Baghdad, Institute of Laser for Postgraduate Studies, Baghdad, Iraq

## Abstract

**Background:**

This study aims to assess the efficacy of Er; Cr: YSGG laser operating under sub-ablative settings for the remineralization of artificially induced white spot lesions on the enamel of primary teeth, both as a standalone treatment and in conjunction with 1.23% acidulated phosphate fluoride (APF) gel.

**Material and Methods:**

Eighty primary posterior teeth were used to induce white spot lesions and were randomly divided into eight groups. The groups consisted of a negative control group (G1), a 0.75W laser irradiation group (G2), a 0.5W laser irradiation group (G3), a 0.25W laser irradiation group (G4), a positive control group with APF gel application (G5), and three groups that received laser irradiation of 0.75W, 0.5W, and 0.25W followed by APF gel application (G6, G7, and G8) respectively. Surface microhardness, SEM, and X-RD were used to evaluate the remineralization effect.

**Results:**

There was a notable enhancement in surface microhardness post-treatment with the laser, APF gel, and their combination compared to the negative control group. The most pronounced increase in surface microhardness was observed in the combination treatment groups (G6, G7, and G8). SEM analysis showed increased surface roughness in groups treated with 0.75W and 0.5W laser power. X-RD assessments indicated an augmentation in crystal intensity within groups G6 and G8.

**Conclusions:**

The combined application of Er; Cr: YSGG laser under sub-ablative parameters and APF gel demonstrated a superior potential for remineralizing primary teeth enamel affected by white spot lesions.

** Key words:**Er;Cr:YSGG laser. Sub-ablative irradiation. Remineralization. White spot lesion. APF gel. SEM. X-RD.

## Introduction

The prevention and treatment of dental caries remains a pressing concern within the field of dentistry, given the implications for public health and human well-being. The development of dental caries is marked by changes in the apatite crystals of the enamel, which can result in the formation of white spot lesions, initial enamel caries, dentin involvement, and cavitation. Despite modern advances in dental care, the prevention and treatment of dental caries continues to be an active area of research worldwide. In the early stages of enamel caries, the appearance of chalky irregularities indicates a disruption in biological mineralization activity, resulting from a loss of mineral content on both the enamel surface and subsurface (1). Contemporary approaches to caries management prioritize non-cavitated carious lesions, with an emphasis on halting or reversing progression and promoting remineralization, rather than resorting immediately to drilling and filling procedures (2).

Fluoride topical treatment is a widely used method for remineralization and stabilization of early carious lesions. Sodium fluoride, acidulated phosphate fluoride, and MI paste plus are commonly employed for this purpose. Among these, acidulated phosphate fluoride gel is the most widely used method for fluoride therapy. This gel reacts with enamel to form calcium fluoride (CaF2), which develops on the enamel surface and subsurface of enamel carious lesions (3). When fluoride is topically applied to white spot lesions (WSLs) *in vivo*, it can be eliminated through various mechanisms such as back diffusion, back exchange, and migration from the mineral to the surrounding tissue fluid, saliva, or plaque fluid (2). However, the amount of fluoride released from the reservoir over time decreases. This approach of topical application of fluoride has drawbacks including the possibility of dental fluorosis, particularly in children (4). Furthermore, the remineralization process may lead to external staining. Besides, this method requires the patient’s consent and takes more time to complete. If superficial remineralization is performed while the lesion is still porous, it may cause unpredictable and persistent white discoloration (5). Early preventive methods are insufficient to prevent new caries lesion development in high-risk individuals (2). In recent years, however, this approach has been challenged by a need for minimally invasive and less traumatic dental caries management and prevention methods for young children. Laser irradiation is one such solution that can increase fluoride uptake (7).

Lasers are increasingly being employed in the prevention of dental cavities due to their noTable impact on dental hard tissue. Laser irradiation has been found to enhance enamel acid resistance by means of fusing, modifying crystallinity, and reducing chemical permeability (8). By melting hydroxyapatite crystals, laser irradiation can recrystallize enamel, thereby improving its resistance to acid and reducing its permeability (2). Additionally, Erbium lasers have demonstrated success in preventing caries by reducing microbe count and changing enamel structure (9), offering a promising approach to maintaining optimal oral hygiene.

The Er. Cr: YSGG laser exhibits peak absorption by hydroxyapatite (HA) and water at 2780nm within the infrared spectrum, rendering it efficacious for a multiple of dental procedures involving both hard and soft tissues (10,11). Irradiation with the Erbium Laser induces modifications in the calcium-to-phosphorus ratio, the carbonate-to-phosphate ratio, and the overall composition of inorganic and organic constituents within hard dental tissues. These alterations influence the tissue’s molecular structure and its vulnerability to acidic degradation and dental caries. Consequently, this implies that laser etching may facilitate remineralization processes by engendering microspaces that entrap free ions, thereby reducing acid damage and caries sensitivity (12).

The research community has been mostly focused on the impact of fluoride and laser on permanent teeth, leaving a gap in our knowledge of their effects on primary teeth. To address this gap, an *in vitro* study was conducted using primary teeth with induced white spot lesions to replicate oral conditions. The study aimed to compare the efficacy of Er, Cr: YSGG laser alone and in combination with APF gel (1.23% F) in demineralizing WSLs. The findings of this study could inform future research and contribute to improving dental care for primary teeth.

## Material and Methods

Ethical approval (19/10/2022-488) was obtained from the Research and Ethics Committee of Institute of Laser for Postgraduate Studies, University of Baghdad.

In this study, a total of 80 primary posterior teeth were selected, which were either extracted for orthodontic reasons or naturally exfoliated. Prior to inclusion in the study, each tooth crown underwent a meticulous examination under a 40X optical microscope (OLYMPUS/BX51, Korea) to exclude any specimens with visible defects such as cracks, cavities, abrasion, fluorosis, or discoloration (13). Initial cleaning involved the removal of tissue remnants and debris using an ultrasonic scaler. Subsequently polishing was performed using fluoride-free pumice (Perfection Plus, Hants, UK), with prophylactic rubber cups. The roots were sectioned 2 mm below the cementoenamel junction (CEJ) utilizing a water-cooled diamond disc.

The buccal surfaces of the teeth were then meticulously polished with Sof-Lex™ discs (3M™ ESPE, USA) attached to a slow-speed handpiece. To ensure sterility, the samples were immersed in a 0.1% chloramine T solution (BDH, England) for a maximum duration of one week, followed by storage in distilled water, which was refreshed on a weekly basis until further use (14,15).

The specimens were embedded in cold-cure acrylic resin cylindrical blocks (Duracryl® Plus, Spofa Dental, Kerr business, Czech Republic), ensuring that only the polished enamel surfaces were exposed. A standardized 3x3 mm section of adhesive tape was applied to each enamel surface, which was then coated twice with an acid-resistant nail varnish (Flormar, Turkey). The tape was subsequently removed to create a uniform window for subsequent procedures (16). The embedded teeth were stored in distilled water at ambient temperature to mitigate the risk of dehydration prior to the demineralization phase.

-Demineralization process:

To induce artificial enamel caries(white spot lesions), a demineralization regimen was employed. The teeth specimens were immersed in a demineralizing solution. This solution was composed of 2.20 mmol/L calcium chloride (CaCl2), 2.20 mmol/L monosodium phosphate (NaH2PO4), 1 mol/L potassium hydroxide (KOH), and 0.05 mol/L acetic acid (CH3COOH). The solution’s pH was maintained at 4.4. The teeth were placed in a light-resistant container within a water bath maintained at 37°C (BS-11/LAB COMPANION, Korea) for four days. Alterations in the enamel surface were monitored and deemed significant when visible changes were detected under wet and dry conditions (17). The presence of demineralization was quantitatively assessed using a DIAGNOdent™ pen 2190 (KaVo, Biberach, Germany), with the mean fluorescence value of three consecutive readings per tooth determining the extent of lesion formation (18).

-Experimental groups:

After inducing white spot lesions (WSLs), each specimen was subjected to a rinse with 10 ml of deionized water and subsequently air-dried using a compressed air stream. The specimens were then allocated into eight experimental groups, each comprising ten samples (n=10), in a randomized manner:

• Group 1 (G1): Served as the negative control, receiving no intervention.

• Group 2 (G2): Received an Er; Cr: YSGG laser at a setting of 0.75 W and 20Hz, with 60% water and 40% air irrigation.

• Group 3 (G3): Received an Er;Cr: YSGG laser treatment at 0.5 W and 20 Hz, with the same irrigation parameters as G2.

• Group 4 (G4): Received an Er;Cr: YSGG laser at 0.25 W and 20 Hz, maintaining identical irrigation conditions as G2 and G3.

• Group 5 (G5): Acted as the positive control group, with samples exposed only to a four-minute application of acidulated phosphate fluoride gel at a concentration of 1.23%.

• Group 6 (G6): Subjected to a combined treatment protocol starting with the Er; Cr: YSGG laser at 0.75 W and 20 Hz (with the previous irrigation settings), followed by a four-minute application of the aforementioned fluoride gel.

• Group (G7): Subjected to a combined treatment protocol starting with the Er; Cr: YSGG laser at 0.5 W and 20 Hz (with the previous irrigation settings), followed by a four-minute application of the aforementioned fluoride gel.

• Group (G8): Subjected to a combined treatment protocol starting with the Er; Cr: YSGG laser at 0.25 W and 20 Hz (with the previous irrigation settings), followed by a four-minute application of the aforementioned fluoride gel.

-Lasing.

An Er; Cr: YSGG laser (2780nm, Waterlase iPlus; Biolase, Irvine, CA, USA) was utilized for the enamel irradiation of the specimens. The laser operated at output powers of 0.25 W, 0.5W, and 0.75W, with a pulse width of 140 µs and a repetition rate of 20 Hz. The irrigation system provided 40% air and 60% water spray. Energy transmission was facilitated through a fiber optic system equipped with a 600µm beam diameter MZ6 sapphire gold tip. The laser was applied from a 1-2 mm distance in a non-contact (H) mode (19). To ensure consistency across all samples, CNC machines were employed to standardize the lasing process (20).

-Application of APF (1.23%):

The enamel surfaces were treated with APF gel (1.23%) (Ionite, Dorado Dental Supply, PR, USA), applying a layer of 1-2 mm thickness using a cotton bud for four minutes. Following the application, the gel was meticulously removed with a cotton roll in accordance with the manufacturer’s guidelines.

Evaluation of enamel surface microhardness: Microhardness testing was conducted using a Vickers microhardness tester (TH715, SN: 0003, Beijing Time High Technology Ltd., China). A force of 1000g (9.8N) was applied for 15 seconds to each sample, and the mean of three measurements was calculated to determine the microhardness value. These measurements served as the baseline following demineralization, laser irradiation, and APF gel (1.23%) application (21).

-Scanning electron microscopy:

Morphological examination of a representative sample from each experimental group was performed using a scanning electron microscope (SEM, TESCAN, VEGA II, Czech Republic) at a magnification of 4000x. Prior to SEM analysis, the samples were sputter-coated with gold (Emitech-K500X, Quorum Technologies, Ashford, UK) to enhance image quality.

-X-Ray diffraction:

X-Ray diffraction (XRD) was employed to elucidate the crystallographic patterns of the enamel surface across all experimental groups. The analysis was conducted using an XRD-6000 (SHIMADZU, Japan) apparatus, operating at 35 kV and 25 mA with CuKa radiation at 10° ≤2 θ ≤70°. PANalytical X’pert HighScore 4.5 used to analyze the results. The peak width was measured by the full width at half maximum. Crystal size determination was executed employing Scherrer’s equation: (D = 0.89 λ/βcosθ) (λ: CuKa wavelength, β: FWHM, θ: Diffraction angle. (22)

Statistical analysis: The data was analyzed using various statistical methods, including descriptive statistics, one-way analysis of variance (ANOVA), Tukey’s Honestly Significant Difference (HSD) test, and Paired t-test. A significance threshold was set at ( *p* < 0.05 ). Analyses were performed using IBM SPSS Statistics software, version 29.

## Results

-DIAGNOdent™ pen readings:

According to the manufacturer’s instructions, specimens with a score between 14-20 were classified as having WSLs. (23)

-Surface microhardness evaluation:

The mean values, standard deviations, and results of the one-way Analysis of Variance (ANOVA) for the microhardness of the study groups at baseline, post-demineralization, and following surface treatment are delineated in  Table 1. The ANOVA revealed an insignificant disparity in microhardness among the study groups at the initial measurement and subsequent to demineralization, with the exception of the treatment cohorts, which exhibited a statistically significant divergence (*P*<0.001)). Employing the paired t-test, a statistically significant alteration in microhardness was observed for all study groups between the baseline and post-demineralization states, as indicated in  Table 2, and between the post-demineralization and post-treatment measurements, as presented in  Table 3. The Tukey’s (HSD) test conducted on the post-treatment study groups revealed an insignificant variation among the treatment groups (G2, G3, G4, and G5). Conversely, the combined treatment groups (G6 and G7) demonstrated a significant discrepancy when compared to the solely laser-treated cohorts (G2, G3, and G4), and an insignificant difference relative to the exclusively fluoride-treated group G5, as depicted in  Table 4.

**Figure 1:**
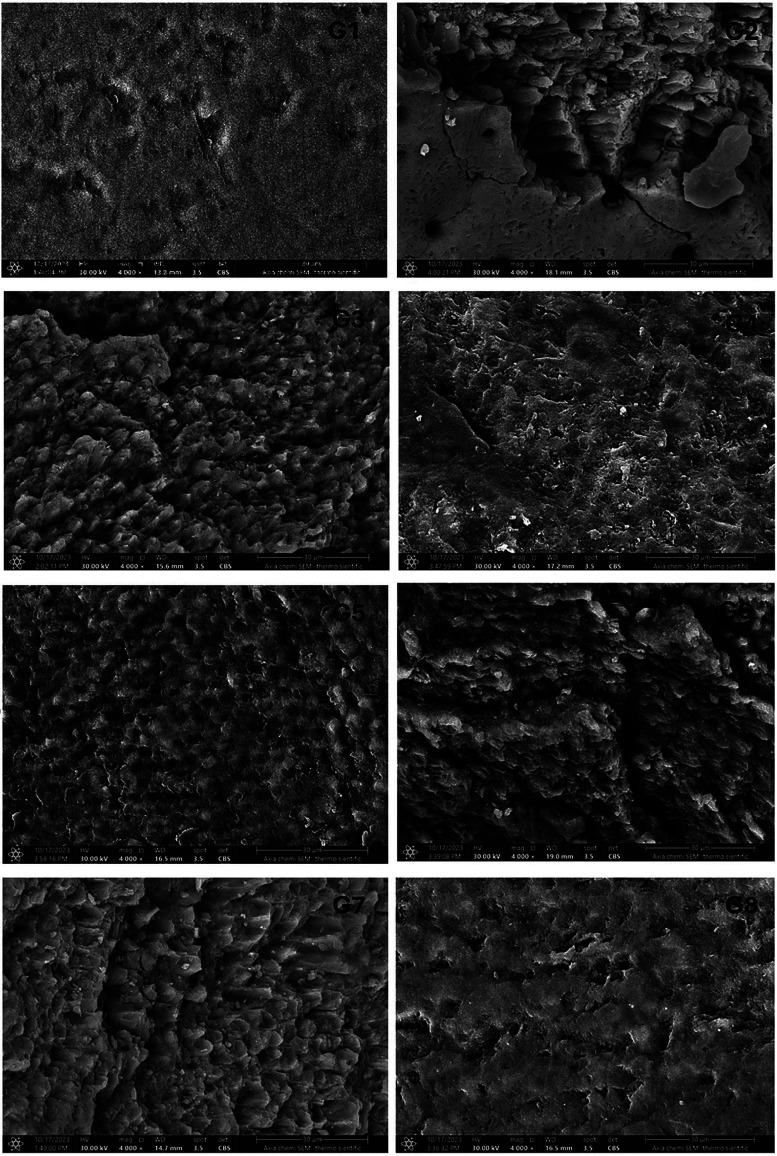
SEM images at magnification × 4.00 k of the study groups’ samples following surface treatment.

-Electron Microscopy (SEM) Observations: Figure 1 elucidates the morphological alterations of enamel samples post-demineralization, laser irradiation, and/or application of APF gel, with SEM images captured at a magnification of 4000x. The control group (G1) presented with degraded enamel surfaces, characterized by rough and etched patterns, alongside a smear layer. In contrast, samples from groups G2 and G3 exhibited distinct imperfections: rough and sharp edges, deep craters, empty interprismatic spaces, and a noTable absence of melting or fusion of the enamel rods, as well as an absence of a smear layer.

Group G4 samples, subjected to laser irradiation at 0.25W, demonstrated melting and fusion of the enamel rods, indicating a transformative effect of the laser treatment. Group G5 was characterized by the formation of a precipitated layer that covered the degraded enamel surface, suggesting a remineralization process. Groups G6 and G7 were notable for the presence of globular precipitates on the enamel prisms and within the inter-prismatic spaces, indicative of significant mineral deposition. Finally, group G8 displayed a higher degree of homogeneous precipitation on the laser-treated enamel surface, with a reduced presence of porosities and irregularities when compared to groups G6 and G7, suggesting an optimized remineralization outcome.

**Figure 2:**
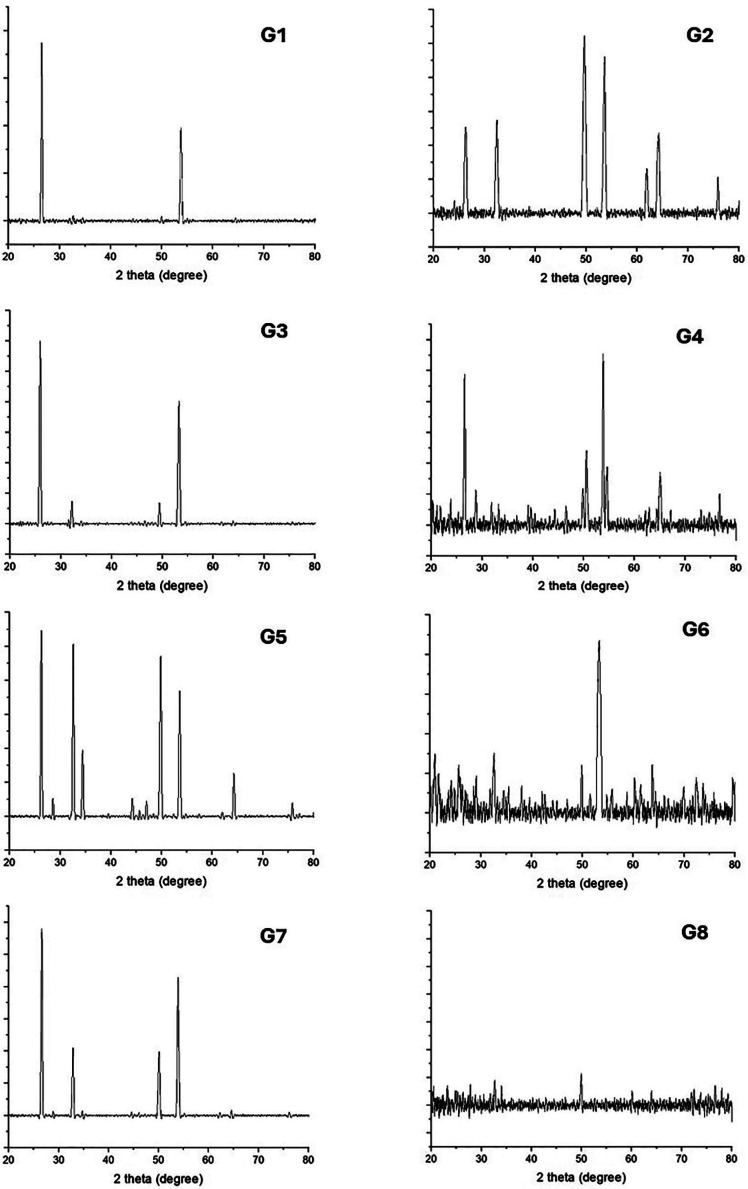
X-RD spectra of study groups’ samples following surface treatment.

X-Ray Diffraction (XRD): XRD spectra (Fig. 2) confirmed hydroxyapatite (HA) as the predominant component on enamel surfaces across all groups, with minimal spectral deviations from the standard HA pattern (JCPDS 01-074-0565). Post-treatment XRD analyses yielded mean intensity (I%), full width at half maximum (FWHM), and crystal size in angstrom (A°), as presented in  Table 5.

## Discussion

Drawing upon the insights from the study by Gouda *et al*. (25), white spot lesions (WSLs) are identified as the initial clinical sign of dental caries, presenting as chalky white opacities. These lesions consist of a porous subsurface enamel, while the external enamel layer remains largely intact and radiopaque. The management of WSLs aims to prevent further demineralization and restore mineralization, thereby reducing lesion activity and improving their visual appearance (17). The application of laser therapy, with or without fluoride, has been explored the researchers as a potential treatment method (24). In this *in vitro* experimental study, we evaluated the efficacy of 1.23% APF gel and Er, Cr, YSGG laser, both independently and in combination, on artificially induced WSLs on the crowns of 80 primary teeth, categorized into eight distinct groups.

Tooth erosion, indicative of mineral loss from dental hard tissues, is accurately assessed by measuring surface microhardness—a reliable indicator of enamel’s mineral content (19). Our study observed a significant reduction in mean microhardness following demineralization (*p*<0.001). To mimic the condition present near the teeth during demineralization *in vivo*, an acetate buffer was utilized for its efficacy in inducing deeper and more rapid formation of WSLs compared to lactate buffer. The incorporation of calcium and phosphate into the acidic buffer system led to a partial saturation, resulting in a surface topography akin to natural WSLs, characterized by an intact exterior surface and subsurface demineralization (18). Additionally, the pH of the demineralization solution was adjusted to 4.4, optimizing the dissolution of hydroxyapatite and fluorapatite crystals, which typically require a pH of 5.5 and 4.5 respectively, as stated by Gouda *et al*. (25).

In the context of this study, the groups treated with laser (G2, G3, and G4) exhibited a significant increase in surface microhardness, comparable to the fluoride-treated group (G5). This suggests the potential of laser technology as a prophylactic treatment for paediatric patients, akin to traditional fluoride use. The application of lasers may offer a more engaging and less difficult experience for children since they are more likely to be interested in an exciting experience. Furthermore, it presents a more streamlined approach for clinicians due to reduced preparatory requirements.

Moreover, the groups receiving combined treatments (G6, G7, and G8) showed an elevated mean microhardness, with a noTable difference from the groups treated solely with laser (G2, G3, and G4), and a non-significant variance from the fluoride-only treated group (G5). This indicates that a combined regimen could be considered as a preventive strategy in patients at high risk for caries. These observations are in agreement with the findings of Assarzadeh *et al*., Adel *et al*., and Molaasadollah *et al*. (2,24,26). Conversely, Tagliaferro *et al*. reported no significant difference between combined laser and fluoride therapy compared to laser treatment alone in enhancing enamel resistance to demineralization in primary teeth, a discrepancy that may be attributed to the type of laser used, as their study utilized a CO2 laser (27). Moslemi *et al*. found that enamel demonstrated increased resistance to demineralization following Er; Cr: YSGG laser exposure and subsequent APF gel treatment, compared to the use of APF gel alone. This might be ascribed to the utilization of permanent teeth in their investigations and the approach employed to evaluate the enamel’s resistance (28). Gouda *et al*. reported that laser irradiation, both alone and in combination with fluoride, significantly increased enamel surface microhardness compared to fluoride-only treatments, a finding that contrasts with our results. This could be due to differences in fluoride agents used, irradiation times, and methods of WSL induction (25). Subramaniam and Pandey’s study revealed that Er;Cr:YSGG irradiation followed by CPP-ACP application resulted in a significant rise in the surface microhardness of primary teeth enamel, surpassing the effect observed with CPP-ACP alone (29). The variations in outcomes observed in our study may be due to the use of different fluoride agents and repeated laser irradiation.

The possible enhancement in the microhardness of the enamel upon laser irradiation can be attributed to the influence of the Er;Cr:YSGG laser operating at a sub-ablative threshold. This, as suggested by Ramlho *et al*., is explained by the laser induction of chemical and crystallographic alterations in dental mineralized tissues, leading to an augmented resistance of enamel. (30). Furthermore, according to Gouda *et al*., additional research has indicated that laser irradiation has the potential to facilitate the creation of micro gaps inside enamel. This, in turn, can enhance the collaboration or diffusion of fluoride within the enamel’s structure, so enabling the construction of a fluoride reservoir and the formation of an adherent CaF2-like material that is crucial for erosion protection (25). Per this study project, Fekrazad and Ebrahimpour suggested an alternative hypothesis posited that the resilience of enamel is achieved by the disintegration of organic matrix proteins and the obstruction of interprismatic gaps (31). To safeguard the vitality of the pulp from potential thermal harm and avoid the formation of undesired chemical phases that are more susceptible to acid breakdown, a water-to-air irrigation ratio of 60% water to 40% air was employed. The selection of laser frequency was also studied in this trial, the frequency of 20Hz was used to ensure sufficient cooling between pulses and this may reduce the thermal deterioration effects of the laser and preserve tooth vitality as also stated by Zamudio *et al*., and Erkmen Almaz *et al*. (12,32). Furthermore, it has been reported by Harashima *et al*., that the enamel absorption coefficient is approximately 50 mm-1 (33). Additionally, Ivanov *et al*., have indicated that the laser beam can break into the superficial layer of the enamel to a depth of roughly 21µm-1, hence conserving the vitality of the pulp (34).

The SEM images of the negative control group G1 exhibited a non-distinctive pattern of eroded enamel surface with a covering of smear layer. An altered morphology with increased surface roughness and enamel rods exposition after irradiation with Er;Cr:YSGG laser particularly with irradiation powers of 0.75W and 0.5W. The increased roughness following laser irradiation agrees with the findings of Gouda *et al*., Malik *et al*., Sun *et al*. (25,35,36). According to Gouda *et al*., the application of laser irradiation to the enamel surface leads to an augmented roughness, resulting in the formation of a retentive niche for fluoride that exhibits enhanced resistance to dissolution. Moreover, according to the findings of Ersahan and Sabuncuglu, the observed increase in roughness on the enamel surface following Eribuim laser irradiation may be attributed to the ablation process, which induces structural modifications resulting from the phase transformation or melting of inorganic compounds, as well as the expansion of the organic matrix. (37). The laser energy that is absorbed is transformed into heat, causing the water within the tooth to boil. This process results in the formation of high-pressure steam, which undergoes explosive vaporization. Consequently, the previously smooth tooth surface transforms into a generally flaky structure with irregular serrations and micro-fissures. This altered structure is typically devoid of carbonization and smear layers (38). Furthermore, the presence of water during irradiation significantly enhances ablation since Erbium lasers have a strong attraction to water. According to Colucci *et al*., as the amount of water increases, the laser’s power on the enamel surface decreases (39).

Hydroxyapatite crystals, fundamental constituents of dental enamel, are susceptible to acid erosion, which can diminish their quantity and hamper their structural integrity (40). This phenomenon may elucidate the enlarged crystal dimensions and reduced mineral density observed in the negative control group G1. According to Akkus *et al*. and Wang *et al*., a reduction in enamel mineral content is associated with increased porosity, thereby heightening the risk of caries and diminishing the enamel’s microhardness (41,42). The X-Ray Diffraction (X-RD) results indicated that the most pronounced mineral density in this study was noted in the combination treatment groups G6 and G8, likely due to the deposition of fluorapatite on the enamel surface. The noted increase in mineral content is interpreted as an enhancement in the enamel’s resistance to demineralization, a key function of fluoride. It is contended by some that fluoride accelerates remineralization, a main mechanism in the prevention of dental cavities (43). Nonetheless, the Full Width at Half Maximum (FWHM) for these groups was the most substantial, as FWHM is inversely proportional to crystallinity (42), indicating a prevalence of more amorphous crystallites, as depicted in Figure 2. Fluoride’s stability on enamel surfaces is attribuTable to the crystals undergoing polarity alterations, leading to an increased absorption and a surge in fluoride following the loss of carbonate and water; this underscores the enhanced efficacy of fluoride therapy when combined with laser irradiation (2).

Variations in irradiation parameters, laser conFigurations, demineralization solutions, and examination protocols may explain the varied outcomes across different studies. Further research is warranted to compare demineralization and remineralization methodologies. Determining the most effective timing for laser application—whether before, after, or in conjunction with fluoride treatment—remains an open question. Additionally, the efficacy of single versus multiple surface treatments merits extended investigation.

## Conclusions

The current research demonstrates that the synergistic application of Er, Cr: YSGG laser irradiation followed by 1.23% acidulated phosphate fluoride (APF) gel substantially outperforms the singular use of laser treatment in promoting the remineralization of white spot lesions (WSLs) on primary teeth enamel.

## Figures and Tables

**Table 1:** Mean, Standard deviation, and one-way ANOVA test of study groups’ microhardness.

Microhardness	Mean ± S.D.	Mean ± S.D.	Mean ± S.D.
Group	Baseline	After demineralization	After treatment
G1	228.25 ± 47.77	185.98 ± 36.08	------------------------------
G2	217.88 ± 28.95	171.61 ± 28.05	201.26 ± 25.1
G3	223.24 ± 26.9	176.14 ± 20.02	212.12 ± 24
G4	228.25 ± 33.6	176.02 ± 27.95	212.69 ± 30.37
G5	202.39 ± 32.04	188.86 ± 33.59	221.88 ± 21.76
G6	226.63 ± 29.98	181.75 ± 30.23	249.71 ± 31.7
G7	223.8 ± 21.63	195.67 ± 24.9	246.18 ± 20.47
G8	223.5 ± 33.92	186.4 ± 34.36	241.07 ± 28.42
F value*	0.112	0.708	19.7
P-value*	0.10	0.67	<0.001
*One-way ANOVA			

*One-way ANOVA

**Table 2:** Paired t-test of the study groups’ microhardness between the baseline and after demineralization.

Microhardness	Baseline	After demineralization	Mean difference	T-Statistic	P-value^1^
Group					
G1	228.3 ± 47.8	186.0 ± 36.1	42.28	5.8	<0.001
G2	217.9 ± 28.9	171.6 ± 28.5	46.27	13.91	<0.001
G3	223.2 ± 26.9	176.1 ± 20.0	56.1	7.06	<0.001
G4	228.3 ± 33.6	176.0 ± 27.9	52.23	9.37	<0.001
G5	222.4 ± 32.0	188.9 ± 33.6	33.53	4.84	<0.001
G6	226.6 ± 30.0	181.8 ± 30.2	44.88	10.52	<0.001
G7	223.8 ± 21.6	195.7 ± 24.9	28.14	6.73	<0.001
G8	223.5 ± 33.9	186.4 ± 34.4	37.09	8.36	<0.001

**Table 3:** Paired t-test of the study groups’ microhardness between the measurements after demineralization and after treatment.

Microhardness	After demineralization	After treatment	Mean difference	T-Statistic	P-value^1^
Group					
G2	171.6 ± 28.5	201.3 ± 25.1	-29.7	-4.59	0.0013
G3	176.1 ± 20.0	212.1 ± 24.0	-36	-8.77	<0.001
G4	176.0 ± 27.9	212.7 ± 30.4	-36.7	-6.83	<0.001
G5	188.9 ± 33.6	221.9 ± 21.8	-33	-4.41	0.0017
G6	181.8 ± 30.2	249.7 ± 31.7	-67.9	-13.33	<0.001
G7	195.7 ± 24.9	246.2 ± 20.5	-50.5	-10.64	<0.001
G8	186.4 ± 34.4	241.1 ± 28.4	-54.7	-12.19	<0.001

**Table 4:** Tukey multiple comparisons of microhardness means (95% family-wise confidence level) after treatment.

Microhardness		After treatment	
I	J	Mean difference (I-J)	Adjusted P- value
G2	G3	-10.86	0.97
	G4	-11.43	0.96
	G5	-20.62	0.57
	G6	-48.45	<0.001
	G7	-44.92	0.01
	G8	-39.81	0.01
G3	G4	0.57	1
	G5	-9.76	0.99
	G6	-37.59	0.02
	G7	-34.06	0.04
	G8	-28.95	0.2
G4	G5	-9.19	0.99
	G6	-37.02	0.03
	G7	-33.94	0.05
	G8	-28.38	0.2
G5	G6	-27.83	0.2
	G7	-24.3	0.3
	G8	-19.19	0.5
G6	G7	3.53	1
	G8	8.64	1
G7	G8	5.11	1

**Table 5:** Mean of intensity %, FWHM, and crystal size of the study samples after treatment.

Group	Intensity %	FWHM (°)	Crystal size Å
G1	24.887	0.31336	371.3
G2	48.4	0.3946	281.09
G3	15.625	0.3557	377.5263
G4	46.455	0.54567	271.6667
G5	32.3672	0.2527	260.833
G6	82.754	0.6409	192.8
G7	22.565	0.3703	323.47
G8	68.05	0.7504	136

## Data Availability

The datasets used and/or analyzed during the current study are available from the corresponding author.
